# Synbiotic Diet Prevents *Escherichia coli* Lipopolysaccharide-Induced Gut Dysbiosis and Intestinal Disruption After Weaning in Piglets

**DOI:** 10.3390/cimb48030298

**Published:** 2026-03-11

**Authors:** Iulian Alexandru Grosu, Valeria Cristina Bulgaru, Daniela Eliza Marin, Ionelia Taranu, Gina Cecilia Pistol

**Affiliations:** Laboratory of Animal Biology, INCDBNA-IBNA, National Research and Development Institute for Biology and Animal Nutrition, 077015 Balotesti, Romania; cristina.bulgaru@ibna.ro (V.C.B.); daniela.marin@ibna.ro (D.E.M.); ionelia.taranu@ibna.ro (I.T.); gina.pistol@ibna.ro (G.C.P.)

**Keywords:** synbiotic diet, piglet intestinal barrier integrity, gut microbiota, LPS challenge, weaning

## Abstract

Post-weaning piglets are vulnerable to intestinal barrier disruption and microbiota imbalance, which can be exacerbated by bacterial endotoxin; this study assessed whether a synbiotic diet based on grape seed and camelina meals plus Lactobacillus probiotics can attenuate an *Escherichia coli* lipopolysaccharide (LPS) challenge. Twenty weaned piglets were randomized (n = 5/group) to control, LPS, synbiotic (SYN), or SYN+LPS diets for 21 days. The control diet consisted of a complete standard corn–soybean-based feed. The SYN diet contained a basal diet with 5% prebiotic mix (grape seed meal–camelina meal) and 0.1% probiotic mix including *Lactobacillus acidophilus*, *Lactobacillus paracasei*, and *Lactobacillus rhamnosus*; on day 21, the LPS and SYN+LPS animals received an LPS challenge and were sampled 3 h later. The expression of colonic genes coding for proteins like tight junctions, mucus/epithelial function, Toll-like receptors and signaling molecules involved in innate response was quantified by quantitative PCR arrays, and the microbiota composition was profiled by 16S rRNA sequencing. The LPS challenge reduced the expression of barrier- and mucus-associated genes and increased that of Toll-like receptors and signaling pathway markers, accompanied by microbial shifts, with reduced beneficial taxa and increased *Megasphaera elsdenii*. The synbiotic diet counteracted these transcriptional and microbial changes. Overall, the synbiotic supported epithelial integrity and moderated innate immune activation during acute endotoxin stress after weaning.

## 1. Introduction

After the EU ban on antibiotics (1 January 2006 under Regulation (EC) No 1831/2003) as growth promoters in farm animal feed due to their excessive use during the weaning period (e.g., pigs), there has been a huge demand for alternatives [1]. In pigs, weaning is a critical phase in which abrupt dietary, environmental, and social changes disrupt intestinal structure and function, predisposing piglets to gastrointestinal disturbances and immune dysregulation and increasing their susceptibility to inflammatory stimuli such as bacterial toxins [2,3]. Lipopolysaccharide (LPS), a major component of the outer membrane of Gram-negative bacteria, is a powerful pro-inflammatory stimulus which produces inflammation and death. LPS activates Toll-like receptors (TLRs), triggering downstream signaling cascades that compromise barrier integrity and alter immune-gene expression, often leading to inflammation [4,5]. Innovative nutritional strategies, including the use of feed additives with probiotics, prebiotics and bioactive compounds such as polyphenols, fiber, vitamins, and minerals from various sources, are among the most promising alternative options to antibiotics [6,7]. These have been intensively investigated over the last two decades as potential replacements for antibiotics.

Studies in the field of probiotics have shown their capacity to improve gastrointestinal health by modulating immune responses, regulating cytokine production, reducing local inflammation, and maintaining epithelial integrity and intestinal barrier function [8,9]. Synbiotic strategies have been reported that attenuate pro-inflammatory signaling pathways such as *NF-κB*. In DSS-treated mice, a synbiotic combining *Limosilactobacillus reuteri* with chitooligosaccharides reduced intestinal inflammation by inhibiting the TLR4/MyD88/*NF-κB* (and NLRP3) signaling cascade while preserving intestinal barrier integrity [10]. Similarly, prebiotics and feed ingredients rich in polyphenols, fiber, and micronutrients have demonstrated anti-inflammatory and antioxidant effects on the host.

They can also support a more favorable gut microbiota by promoting beneficial taxa and limiting the proliferation of opportunistic pathogens [11]. Synbiotics, combinations of prebiotics and probiotics, may exert enhanced benefits through synergistic mechanisms.

Research has shown that synbiotics not only support animal health but also promote the growth of beneficial bacteria such as *Bifidobacteria* and *Lactobacilli* [9,12]. Moreover, synbiotics have shown the ability to reduce the adhesion of pathogenic *Escherichia coli* to the intestinal mucosa and inhibit antibiotic-resistant strains in piglet trials [13,14].

Recent interest has turned toward agro-industrial by-products as functional feed due to their rich content of polyphenols, tannins, fatty acids and dietary fiber with antimicrobial and immunomodulatory effects [15,16,17]. By-products such as grape seed meal and camelina meal are abundant sources of phenolic compounds and unsaturated fatty acids and have demonstrated the potential to inhibit pathogenic bacteria, modulate gut microbiota, and strengthen intestinal barrier function [18,19]. Additionally, these compounds may reduce oxidative stress and inflammation, contributing to improved resilience in piglets facing post-weaning challenges [19,20].

Grape seed meal and camelina meal were selected over other by-products because they are locally available and offer complementary bioactive profiles like polyphenols and fiber from grape seed meal together with PUFA-rich camelina meal, supporting a regionally sourced and circular economy synbiotic formulation. Our previous in vivo studies demonstrated that grape pomace exerts strong anti-inflammatory activity and has shown antioxidant and gut-protective effects in piglets after weaning [8,21].

In this context, the present study aimed to evaluate the efficacy of a synbiotic diet comprising grape seed meal, camelina meal, and a probiotic mixture of *Lactobacillus* strains in the prevention of *Escherichia coli* LPS-induced gut dysbiosis and the disruption of the epithelial barrier and of the immune response in weaned piglets. LPS is frequently employed in experimental models to induce controlled inflammation and simulate microbial-associated intestinal injury [22]. LPS activates Toll-like receptors (TLRs), triggering downstream signaling cascades which compromise barrier integrity and alter immune-gene expression, often leading to dysbiosis [4]. We assessed the impact of synbiotic dietary intervention on the gut microbiota composition using high-throughput 16S rRNA gene sequencing as well as the expression of genes associated with innate immunity, epithelial integrity, and regulatory signaling pathways in the colon, one of the important sites affected by *Escherichia coli* infections post-weaning [23]. This approach provides insight into the potential use of synbiotics as sustainable alternatives to antibiotics for maintaining intestinal health during the post-weaning period.

## 2. Materials and Methods

### 2.1. Experimental Diets

The two experimental diets were based on corn (57.43%—control diet and 52.67%—SYN diet) and soybean meal (20.0%—control diet and 18.7%—SYN diet, respectively).

The two diets (control and SYN diet) were formulated to meet the nutritional requirements of weaned piglets as indicated by the National Research Council (NRC-2012) [24] and were similar in metabolizable energy (13.60 vs. 13.57 MJ/kg) and crude protein (18.15% vs. 18.12%), with comparable essential amino acid and mineral targets (e.g., total lysine 1.20% vs. 1.21%, methionine + cystine 0.75%, calcium 0.75%, total phosphorus 0.65% vs. 0.66%). The grape seed meal and camelina meal were purchased as dried materials from local suppliers (S.C. OLEOMET S.R.L., Giurgiu, Romania, and Savoarea Soarelui, Oradea, Romania). The two ingredients were mixed in several ratios and chemically analyzed; the 3 to 1 ratio was found to have the highest concentration of bioactive compounds, especially polyphenols. They were included in the SYN diet to a proportion of 5%.

Three *Lactobacillus* strains, *L. acidophilus* (ID 11692), *L. paracasei* (ID 13239), and *L. rhamnosus* (ID IBNA02), were cultured aerobically for 18 h in a BIOSTAT MD bioreactor (B. Braun Biotech, Melsungen, Germany). The bacterial biomass was microencapsulated by spray drying using a Niro Mobile Minor unit (GEA, Düsseldorf, Germany) with maltodextrin, D-glucose, and casein as carriers. The strains were blended in equal proportions (1:1:1), following a protocol previously used by Pistol et al. [25], and added to the feed at 1.5 × 10^7^ CFU/g. The final inclusion rate of the probiotic mix was 0.1%, based on published recommendations [25]. Viable bacterial counts (CFU/g) were determined after spray drying and once again after incorporation into the experimental diets to confirm viability following processing.

The three *Lactobacillus* strains from the probiotic component were administered together as a fixed-dose mixture; single-strain treatment groups were not included as such strain-specific effects cannot be distinguished in this study.

The feed samples were analyzed at the beginning of the trial for their crude protein, fat, fiber, and ash content using the Weende method (ASRO-SR EN ISO 2171:2010, 2010) [26]. The PUFA levels were measured by gas chromatography, while the total and individual polyphenols were quantified using Folin–Ciocalteu and HPLC-DAD-MS methods, respectively, as described by Taranu et al. [27]. The antioxidant capacity was determined following the method of Garcia et al. [28]. Details of the diets’ composition have been reported previously by Pistol et al. [25].

### 2.2. Animals and Experimental Design

Twenty piglets (7.5 ± 0.2 kg, 21-day-old) belonging to the cross-bred TOPIGS-40 hybrid [♀ Large White × Hybrid (Large White × Pietrain) × ♂ Talent, mainly Duroc] were used in the present study. The experiment started immediately after weaning and lasted three weeks (21 days). Animals were distributed into four pens with five piglets per pen, corresponding to the experimental groups (1—control, 2—LPS, 3—SYN, and 4—SYN+LPS) and were housed at the experimental base of the National Research–Development Institute for Biology and Animal Nutrition (IBNA), Balotești, Romania. Each piglet was considered an experimental unit, meaning there were five experimental units per dietary group. A post hoc sensitivity analysis for the one-way ANOVA design (4 groups; n = 5/group; α = 0.05) indicated that the study had 80% power to detect a large omnibus effect (Cohen’s f ≈ 0.84). As such, the experiment was powered to detect large treatment effects. Feed and water were provided *ad libitum* throughout the 21-day study. The LPS challenge was induced in groups 2 and 4 (LPS and SYN+LPS) using an intraperitoneal injection of *Escherichia coli* lipopolysaccharide (80 µg LPS/kg body weight) on day 21. The clinical signs (weight loss, diarrhea, general condition) and treatment performance were monitored as described by Pistol et al. [25]. The experimental protocol was approved by the Ethical Committee (no. 4406/2023) of INCDBNA Balotesti and the animal handling was done in accordance with rules for the handling and protection of animals used for experimental purposes with EU Council Directive 98/58/EC and Romanian Law 43/2014 and in compliance with the ARRIVE guidelines [29]. Three hours after the LPS challenge, the animals were euthanized, and colon samples and colon contents were collected and stored at −80 °C for further analyses. The animals did not receive feed before euthanasia.

### 2.3. qPCR Array Analysis

The total RNA was obtained from colon tissue samples using the RNeasy Plus Universal Mini Kit (QIAGEN GmbH, Hilden, Germany), following the manufacturer’s instructions. The complementary DNA (cDNA) was synthesized from total RNA with the M-MuLV reverse transcriptase kit (Thermo Fisher Scientific, Waltham, MA, USA), according to the manufacturer’s protocol. RNA integrity was assessed using an Agilent Bioanalyzer, and only samples with RIN ≥ 7.0 were used for downstream gene expression analyses.

The gene expression of 31 key genes involved in intestinal inflammatory response and integrity was assessed using the ExProfile™ Gene qPCR Array (GeneCopoeia, Rockville, MD, USA), a customized 96-well plate (Table S1—Supplementary File). For each well, 25 ng of cDNA template containing lyophilized primers was mixed with 10 µL SYBR Green qPCR Master Mix (Promega, Madison, WI, USA) and nuclease-free water to a final volume of 20 µL, as recommended by the manufacturer. Real-time PCR was performed using the cycling protocol described by Pistol et al. [25].

The threshold cycle (Ct) values were normalized against two reference genes, ACTB and GAPDH, which were selected from a panel of five housekeeping genes based on their high expression stability. This selection improved the normalization of the results and reflected the constant expression of the reference genes across all samples, as described by Pistol et al. [25]. The results were expressed as relative fold change (Fc) compared with the control group.

### 2.4. DNA Extraction and 16S rRNA Sequencing

The colonic content DNA was extracted using the QIAamp DNA Stool Mini Kit (QIAGEN, Germany). Negative extraction controls (NECs; extraction blanks without biological material) were included alongside samples during DNA extraction and carried through the downstream workflow to monitor potential reagent and laboratory contamination. The NECs had no amplification signal. DNA integrity and concentration were verified with an Agilent Bioanalyzer. The V3-V4 region of the bacterial 16S rRNA gene was amplified and sequenced on the Illumina PE250 platform by Novogene (Cambridge, UK). The reads were processed using QIIME2 (v2022.2), clustered into operational taxonomic units (OTUs) at 97% similarity, and taxonomically assigned using the SILVA reference database.

### 2.5. Microbiota Analysis

Alpha diversity (Chao1 index, observed OTUs, phylogenetic diversity) and beta diversity (unweighted and weighted UniFrac distances) were computed in QIIME2. Principal coordinates analysis (PCoA) was used to visualize microbial community differences between groups. Taxonomic composition at the phylum and genus levels was derived from OTU tables, and the relative abundances were calculated. The differences in diversity and composition were compared between experimental groups.

### 2.6. Statistical Analysis

The data are presented as mean ± SEM, and each piglet was considered an experimental unit. For the univariate endpoints, differences among the four experimental conditions (control, LPS, SYN, SYN+LPS) were tested by one-way ANOVA (StatView 6.0, SAS Institute, Cary, NC, USA); when the ANOVA was significant, the resulting means were compared using Tukey’s post hoc test. The normality and homoscedasticity were checked prior to ANOVA, and *p* < 0.05 was considered significant. The qPCR array results (31 genes) were additionally explored by principal component analysis (PCA) using XLSTAT. Microbiota analyses were conducted in QIIME2 and R (version 3.5.2), and between-group community differences were assessed using PERMANOVA on the relevant distance matrices.

Microbiota analyses were conducted using QIIME2 and R (version 3.5.2). Beta diversity was calculated using Bray–Curtis dissimilarity and UniFrac distances (weighted and unweighted), and between-group differences in community structure were assessed by PERMANOVA using 999 permutations.

PCA was applied to the 31-gene expression dataset to reduce dimensionality and to visualize whether the overall transcriptional profile clustered by experimental condition, thereby providing an integrative view that complements the gene-by-gene one-way ANOVA and post hoc comparisons.

PCoA was applied to the microbiota beta diversity distance matrices (Bray–Curtis and weighted/unweighted UniFrac) to summarize multivariate community dissimilarities and visualize between-group separation, while formal inference on community-structure differences was performed using PERMANOVA.

## 3. Results

### 3.1. Prebiotics and Diet Composition

The prebiotic mix from SYN diet had a total polyphenol content of 3267.9 mg GAE/100 g feed and a higher antioxidant activity (DPPH) (52.45%) compared to the control. The mix was rich in flavonoids (catechins: 104.70 mg/100 g, caffeic acid: 21.75 mg/100 g, gallic acid: 12.50 mg/100 g, etc), carbohydrates (sucrose: 1063.88 mg/100 g, glucose: 446.15 mg/100 g and fructose: 691.52 mg/100 g) and organic acids (succinic acid: 598.51 mg/100 g; tartaric acid: 231.80 mg/100 g and oxalic acid: 216.63 mg/100 g).

A higher level of PUFA in the SYN diet in comparison with the control diet ((55.56 g/100 g) vs. control diet (50.37 g/100 g)) was also observed. A prevalence of ω-6 PUFAs was found (52.34 g/100 g in SYN diet vs. 47.96 g/100 g in control diet), with an increased concentration of cis-linoleic acid (52.14 mg/100 g in SYN diet vs. 47.38 g/100 g in control diet). A higher content of fiber in SYN diet than in control diet (4.59% vs. 3.7%) was also registered.

The proximate analyses (Weende) showed the isoproteic and isoenergetic composition of the control and SYN diets: the crude protein (18.15% vs. 18.12%) and the metabolizable energy (13.60 vs. 13.57 MJ/kg).

### 3.2. Animal Performances

No differences (*p* > 0.05) were found between the SYN diet and the control diet concerning the average daily gain (ADG), average daily feed intake (ADFI), and feed conversion ratio (FCR). The initial body weight was similar in all experimental groups (control group: 7.4 ± 0.4 kg, LPS group: 7.5 ± 0.4 kg, SYN group: 7.5 ± 0.4 kg, SYN+LPS group: 7.6 ± 0.4 kg). The final body weight recorded at the end of the experiment was not affected by the SYN diet or by the LPS challenge, with the differences being not statistically significant (control group: 11.7 ± 0.5 kg, LPS group: 11.6 ± 0.4 kg, SYN group: 11.7 ± 0.7 kg, SYN+LPS group: 12.2 ± 0.7 kg, *p* > 0.05 in all comparisons). The average daily gain (ADG) was also not different between the experimental groups (205 g/pig/d, 195 g/pig/d, 202 g/pig/d, and 220 g/pig/d for control, LPS, SYN, and SYN+LPS, respectively).

### 3.3. Gene Expression

#### 3.3.1. Effect on Tight-Junction Gene Expression

Tight-junction proteins (Claudin family, occludin, zonula-1 and JAM-A proteins) are important in the regulation of the paracellular transport of substances, including pathogens, and the maintenance of intestinal epithelial barrier integrity. In this study, LPS administration significantly downregulated the expression of all assessed tight-junction genes (Table 1), indicating a disruption of the intestinal barrier. Compared to the control group, LPS reduced *CLDN2* expression 0.14-fold (*p* = 0.001), *CLDN4* 0.62-fold (*p* = 0.013), *CLDN14* 0.46-fold (*p* = 0.001), *CLDN20* 0.33-fold (*p* = 0.0001), occludin (*OCLN*) 0.62-fold (*p* = 0.026), *ZO-1* 0.64-fold (*p* = 0.004), and *JAM-A* 0.57-fold (*p* = 0.005), with no effect on the *CLDN1*, *CLDN5* and *CLDN23* genes (Table 1). The synbiotic diet markedly upregulated 9/10 of the tight-junction genes, except the *CLDN5* gene. The largest increases were observed for *ZO-1* (7.17-fold, *p* = 0.001 vs. control) and *CLDN4* (3.9-fold, *p* = 0.014), followed by *OCLN* (2.2-fold, *p* = 0.014) (Table 1).

In the SYN+LPS group, all the genes affected by the LPS challenge were restored to the control level (Table 1); these results suggest that synbiotic treatment counteracted LPS-induced barrier disruption.

#### 3.3.2. Effect on Barrier-Function Gene Expression

*MUC2* and *ECM*-related genes contribute to the physical and structural components of the gut barrier. In the present study, LPS treatment significantly downregulated *MUC2* expression to 0.65-fold the control (*p* = 0.004), suggesting mucus-layer impairment (Table 1). The synbiotic diet significantly increased *MUC2* expression by 2.56-fold compared to the control (*p* = 0.029), indicating enhanced mucus production and intestinal barrier protection. When administered in combination with LPS, the synbiotic restored *MUC2* levels (1.44-fold vs. control, *p* = 0.032), counteracting the suppressive effect of LPS (Table 1). In contrast, *ECM1* gene expression was not significantly altered by LPS (0.74-fold vs. control, *p* = 0.363). However, the synbiotic diet significantly upregulated *ECM1* expression (1.57-fold vs. control, *p* = 0.012). The SYN+LPS treatment resulted in *ECM1* expression comparable to the control (0.98-fold, *p* = 0.881) (Table 1). These findings support a protective effect of the synbiotic diet on the functionality of the intestinal barrier under LPS challenge.

#### 3.3.3. Tight-Junction Gene Regulators

Tight-junction gene regulators are key determinants of epithelial barrier function, representing *MAGI2* (Membrane-Associated Guanylate Kinase, WW and PDZ Domain Containing 2), *GNAI2* (Guanine nucleotide-binding protein G(i) subunit alpha-2) and *MYO9B* (myosin IXB).

LPS administration significantly downregulated the *GNAI2* (0.61-fold decrease; *p* = 0.006) and *MYO9B* (0.68-fold decrease; *p* = 0.008) genes compared to the control group, indicating a loss of epithelial cohesion, polarity-supporting signaling and cytoskeletal coupling of junctions, respectively (Table 2). By contrast, *MAGI2* expression remained unchanged in the LPS group compared to the control, suggesting that this junctional scaffolding component is relatively resistant to LPS-induced disruption.

Synbiotics alone significantly upregulated *GNAI2* (1.59-fold vs. control, *p* = 0.0313), while *MYO9B* showed a modest but non-significant increase (1.15-fold, *p* = 0.0804). These effects point toward a reinforcement of junctional adhesion and polarity pathways with a trend toward enhanced actin–junction dynamics (Table 2).

In the SYN+LPS group, the expression of *GNAI2* returned to levels comparable with the control group (*GNAI2*: 0.96-fold, *p* = 0.8357), indicating a full normalization of expression. *MYO9B* expression remained slightly suppressed (0.83-fold, *p* = 0.0017 vs. control), suggesting normalization of adhesion and signaling under inflammatory stress but incomplete recovery of myosin IXB-mediated cytoskeletal support (Table 2). Overall, these results support the beneficial role of a synbiotic diet in preserving epithelial gene expression under inflammatory stress.

#### 3.3.4. Effect on Toll-like-Receptor Gene Expression

Toll-like receptors (TLRs) are key components of the intestinal innate immune system. Their altered expression often reflects inflammatory activation. In the present study, lipopolysaccharide (LPS) administration upregulated almost all of the analyzed colonic *TLR* genes (except for TLR-9), with *TLR4*, *TLR6*, and *TLR2* being the most affected (Table 3). Specifically, *TLR4* expression increased 2.74-fold (*p* = 0.0008 vs. control), *TLR6* increased 2.12-fold (*p* = 0.002 vs. control), and *TLR2* showed a 1.93-fold elevation (*p* = 0.010 vs. control) (Table 3). *TLR8* expression was also significantly elevated (1.87-fold, *p* = 0.021 vs. control). Dietary synbiotic administration alone did not significantly affect any of the TLRs compared to the control (*p* > 0.050 for all genes). Importantly, the combination of synbiotic and LPS (SYN+LPS) reversed the LPS-induced upregulation of *TLR4*, which was significantly reduced below control level (0.71-fold vs. control, *p =* 0.022), and numerically reduced *TLR8* expression (0.7-fold vs. control, *p* = 0.051) (Table 3). Expression of *TLR2* and *TLR6* was also numerically decreased under SYN+LPS treatment compared to the LPS group, but these effects were not statistically significant (*p* > 0.050).

These findings suggest that the synbiotic diet modulated the LPS-induced alterations in TLR expression, normalizing receptor levels towards those of controls.

#### 3.3.5. Effect of Dietary Treatments on the Genes Encoding Signaling Mediators

To further explore downstream immune signaling responses, the expression of several key components of the TLR pathway was analyzed (Table 4). LPS administration strongly upregulated *MyD88* and *MD2*, with *MyD88* increasing by 6.7-fold (*p* = 0.006 vs. control) and *MD2* by 3.75-fold (*p* = 0.002 vs. control), confirming a robust activation of *TLR*-mediated inflammatory signaling. LPS also elevated *NF-κB/p65* (2.3-fold vs. control) and *TRAF6* (2.1-fold vs. control) (Table 4).

A synbiotic diet alone did not significantly modify the expression of *MD2*, *MyD88*, or *TRAF6* compared to the control, but was associated with a pronounced reduction in *NF-κB/p65* transcript levels. When administered in conjunction with LPS, the synbiotic largely normalized *MD2* and *MyD88* expression to control values and markedly reduced both *TRAF6*, and *NF-κB/p65*, with transcript levels in the SYN+LPS group falling below those of the control group (Table 4).

Taken together, these findings indicate that the synbiotic exerts a broad modulatory effect on TLR signaling under inflammatory conditions, not only attenuating the LPS-induced upregulation of *MD2* and *MyD88* but also dampening downstream *NF-κB-TRAF6* axis activity.

### 3.4. Gut Microbiota Composition

#### 3.4.1. The Effect of Dietary Treatments on Alpha Diversity in Colon Content of Weaned Piglets

**Figure 1:**
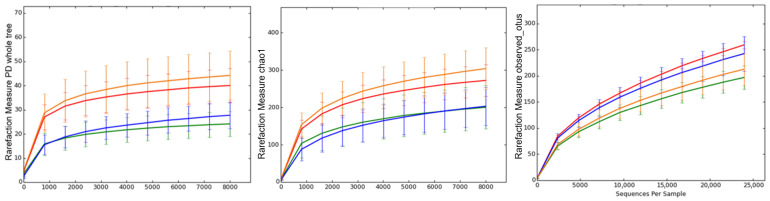
Rarefaction curves of colonic microbiota from weaned piglets showing alpha diversity indices (Chao1, Observed OTUs, and Faith’s Phylogenetic Diversity) across experimental groups: C—control group (red), LPS—LPS group (green), SYN—synbiotic group (blue), SYN+LPS—synbiotic with LPS (yellow). Error bars indicate standard error of the mean (SEM). Each treatment group had 5 piglets (n = 5/group).

Alpha diversity, which reflects the microbial richness and phylogenetic complexity within each group, varied in response to both the LPS challenge and synbiotic diet. The control group consistently displayed a higher alpha diversity across all three metrics, Chao1, observed OTUs, and Faith’s Phylogenetic Diversity, indicating a richer and more complex microbial community (Figure 1). This suggests a stable and healthy gut ecosystem in piglets not exposed to inflammatory or dietary stress.

In contrast, the LPS group showed a visible reduction in alpha diversity across all indices, pointing to a loss of microbial richness and phylogenetic breadth. This decline suggests that the LPS challenge may have disrupted the microbial balance in the colon, potentially reducing microbial resilience and increasing susceptibility to opportunistic colonizers (Figure 1).

Piglets receiving synbiotics (SYN group) exhibited levels of alpha diversity that appeared closer to those of the control group. This suggests that the synbiotic may support a more diverse microbiota, potentially contributing to gut stability in the absence of inflammatory stress.

In the SYN+LPS group, alpha diversity levels were intermediate between those of the LPS and SYN groups. While richness and phylogenetic diversity did not return fully to control levels, the trend indicates a possible preservation of microbial complexity in the presence of synbiotic diet, even under LPS challenge (Figure 1).

These patterns suggest that a synbiotic diet may mitigate the loss of microbial diversity associated with LPS-induced dysbiosis, although further statistical analysis is needed to confirm these effects.

#### 3.4.2. The Effect of Dietary Treatments on Beta Diversity in Colon Content of Weaned Piglets

**Figure 2:**
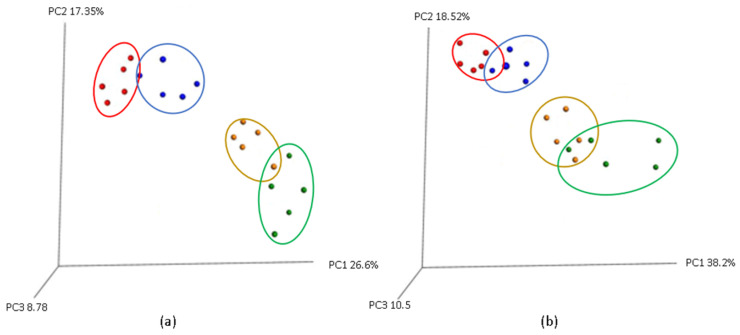
(**a**) Unweighted and (**b**) weighted UniFrac plots. C—control group (red), LPS—LPS group (green), SYN—synbiotic group (blue), SYN+LPS—synbiotic with LPS (yellow). Each point represents one piglet sample; n = 5 piglets per group.

Beta diversity, which evaluates differences in microbial community composition between treatment groups, was analyzed using both weighted and unweighted UniFrac distances (Figure 2). These metrics account for phylogenetic relationships and allow detection of community shifts driven by treatment. Principal coordinates analysis (PCoA) plots based on these distances revealed visible clustering patterns that reflected the influence of LPS and the synbiotic diet on the gut microbiota structure.

The control group formed a distinct cluster, suggesting a stable and consistent microbial composition in piglets not exposed to inflammatory or dietary interventions (Figure 2). In contrast, the LPS-treated group exhibited clear separation from the control group in both weighted and unweighted PCoA plots, indicating that LPS administration induced marked alterations in microbial community structure. This shift likely reflects a disruption in the gut microbial balance, consistent with the pro-inflammatory effects of LPS.

The SYN group appeared to cluster closely with the control group, particularly in the unweighted UniFrac plot, suggesting that synbiotic inclusion preserved a microbial composition similar to that of healthy animals. This observation implies that the synbiotic maintained core microbial populations and limited community divergence (Figure 2).

In the SYN+LPS group, the PCoA plots showed an intermediate position between the SYN and LPS groups (Figure 2). While not fully preventing the alteration of the microbial community induced by the bacterial toxin, the synbiotic appeared to mitigate the LPS-induced shift in microbial structure. This partial overlap suggests a protective effect, whereby synbiotic administration attenuated the extent of LPS-associated dysbiosis and helped preserve aspects of microbial community integrity.

#### 3.4.3. Effect of Dietary Treatments on the Colonic Microbiota at the Phylum Level

**Figure 3:**
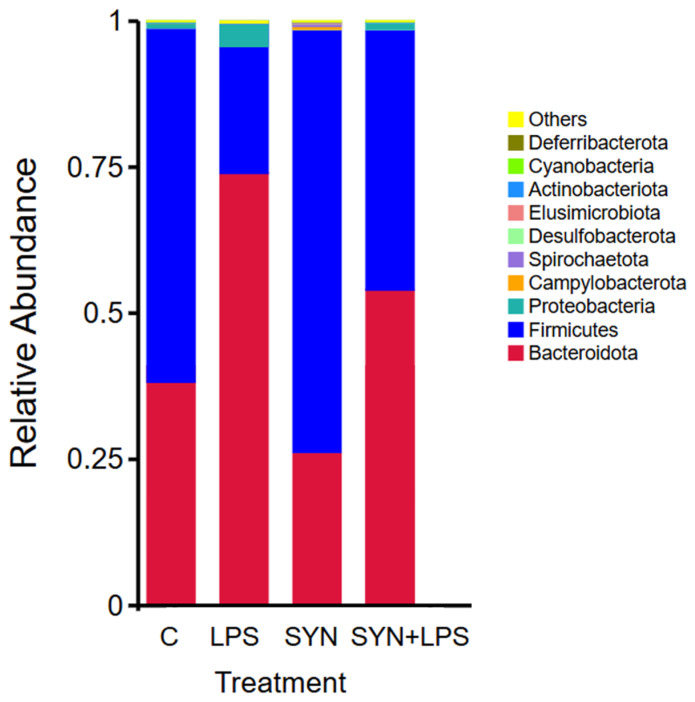
Relative abundance of bacterial phyla in colonic content of weaned piglets across experimental groups: C—control group, LPS—LPS group, SYN—synbiotic group, SYN+LPS—synbiotic with LPS. Relative abundances are shown for n = 5 piglets per group.

The relative abundances of dominant bacterial phyla in the colonic content of weaned piglets were markedly influenced by both LPS challenge and synbiotic diet. The Firmicutes-to-Bacteroidota (F/B) ratio, a key indicator of gut microbial balance, varied substantially across experimental groups. The control group presented an F/B ratio of 1.15, indicative of a balanced microbiome (Figure 3).

In contrast, LPS administration reduced the Firmicutes and increased the Bacteroidota, producing an inverted F/B ratio of 0.35. This shift reflected a profound imbalance and suggested the onset of dysbiosis. Synbiotics alone enhanced Firmicutes abundance, resulting in a ratio of 1.73, consistent with a stable and resilient microbial community. In the SYN+LPS group, the ratio recovered toward equilibrium, reaching 0.97, indicating that the synbiotic partially restored the LPS-induced imbalance (Figure 3).

At the phylum level, the Firmicutes and Bacteroidota dominated all groups but shifted differently according to treatment. In the control group, Firmicutes accounted for 50.9% and Bacteroidota for 44.3%. LPS markedly reduced Firmicutes to 25.7% (a 49% decrease compared to control) and concomitantly increased Bacteroidota to 74.0% (a 67% rise), confirming its disruptive effect. Synbiotics alone increased Firmicutes to 62.0% (a 22% rise over control) and reduced Bacteroidota to 35.9% (a 19% decrease), indicating a modulatory effect on microbial balance. Importantly, in the SYN+LPS group, Firmicutes rose to 42.6% and Bacteroidota decreased to 44.0% compared with the LPS group, representing a 66% increase in Firmicutes and a 40% reduction in Bacteroidota, thereby partially reversing the LPS-induced dysbiosis (Figure 3). Proteobacteria, typically associated with gut instability, expanded under LPS conditions, reaching 3.2% compared to 0.45% in the control (a seven-fold increase).

The synbiotic maintained low levels of Proteobacteria (0.27%), while in the SYN+LPS group, Proteobacteria were reduced to 0.21%, indicating a strong protective effect against LPS-driven expansion.

The minor phyla also displayed treatment effects. Campylobacterota remained stable in the control (0.35%) and LPS groups (0.33%) but decreased sharply under SYN+LPS conditions (0.10%), suggesting a suppressive effect of the synbiotic. Spirochaetota increased three-fold in the LPS group (1.0%) compared to control (0.32%), but decreased to 0.18% with synbiotics alone and to 0.87% with SYN+LPS, indicating partial mitigation. Actinobacteriota, although low in abundance, dropped almost completely in the LPS group (0.01%) but were enriched under SYN+LPS conditions (1.02%), representing a marked increase and suggesting a restorative role of the synbiotic for this phylum (Figure 3).

**Figure 4:**
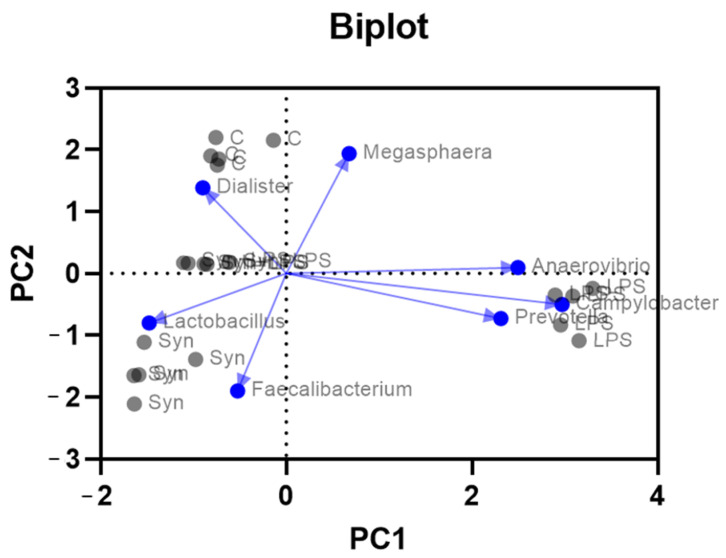
Biplot of colonic microbiota at the phylum level. Gray dots represent individual piglets labeled by treatment (C—control group, LPS—LPS group, SYN—synbiotic group, SYN+LPS—synbiotic with LPS). The first two ordination axes explain 31% (PCoA1) and 22% (PCoA2) of the variance, respectively. Blue dots with arrows show phylum loadings, with arrow length indicating contribution to the ordination. Gray dots represent individual piglet samples.

The PCoA biplot at the phylum level (Figure 4), based on Bray–Curtis distances, showed a clear separation among treatments, with loadings indicating the main taxa-driving differences. The first two axes explained 31% and 22% of the variance in community dissimilarity (53%), showing that the 2D ordination captures a substantial portion of between-sample differences, while additional variation is distributed across higher axes. control and inclusion pigs clustered together on the negative side of PC1 in the direction of Firmicutes, consistent with a stable community dominated by this phylum. LPS samples shifted to the positive side of PC1 toward Proteobacteria, Bacteroidota, and Campylobacterota loadings, indicating an LPS-associated enrichment of these phyla and disruption of community structure.

Overall, the loading pattern supports a stabilizing effect of the dietary treatment under inflammatory challenge.

The LPS + synbiotic group occupied an intermediate position, displaced away from the Proteobacteria and Campylobacterota vectors and closer to the Firmicutes axis, suggesting that inclusion partially countered the LPS-induced shift without fully restoring the control profile (Figure 4).

Together, these findings demonstrate that LPS induces a strong dysbiotic shift at the phylum level, characterized by Bacteroidota expansion, Firmicutes depletion, and Proteobacteria overgrowth.

The synbiotic diet counteracted these effects, restoring the Firmicutes and Bacteroidota proportions closer to equilibrium and reducing potentially harmful phyla, thereby supporting the maintenance of a balanced microbial community in weaned piglets under LPS-induced stress.

#### 3.4.4. Effect of Dietary Treatments on Colonic Content Microbiota at the Genus Level

**Figure 5:**
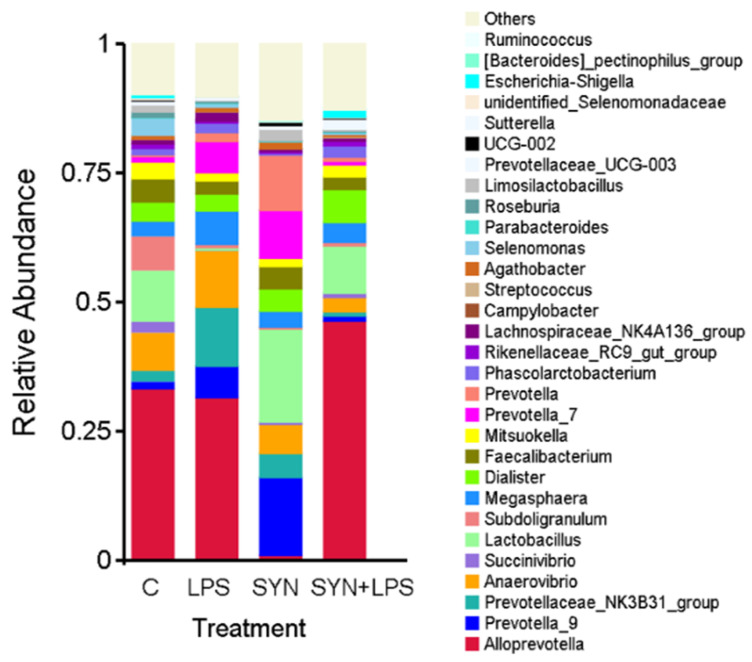
Relative abundance of gut microbiota at the genus level under treatments: C—control group, LPS—LPS group, SYN—synbiotic group, SYN+LPS—synbiotic with LPS. Relative abundances are shown for n = 5 piglets per group.

At the genus level (Figure 5), LPS administration induced significant dysbiosis in the colonic microbiota, as shown by marked shifts in the relative abundance of key genera. Inclusion o the novel feed ingredient modulated these alterations, supporting the growth of beneficial bacteria and mitigating the expansion of taxa associated with inflammatory conditions.

*Faecalibacterium*, a genus linked to anti-inflammatory functions and gut homeostasis, was reduced by 35.2% in the LPS group compared to the control (*p* < 0.01), highlighting its vulnerability to inflammatory stress (Figure 5). In contrast, the synbiotic group showed a 2.4-fold increase in *Faecalibacterium* relative to the control (*p* < 0.001), indicating that the genus was strongly promoted by the dietary intervention. In the SYN+LPS group, *Faecalibacterium* abundance increased 3.1-fold compared to the LPS group alone (*p* < 0.01), suggesting that the feed additive counteracted the LPS-induced depletion and helped restore microbial balance.

*Lactobacillus* levels (Figure 5) dropped by 96.5% in the LPS group versus control (*p* < 0.001), confirming the adverse impact of LPS on this genus, which is crucial for maintaining gut barrier integrity and lactic acid production. In the LPS + synbiotics group, *Lactobacillus* abundance was 48.6% lower than the control (*p* > 0.05), suggesting a moderate, non-significant reduction. However, in this group, levels of *Lactobacillus* were 4.3 times higher than in the LPS group (*p* = 0.02), indicating that the dietary treatment managed to maintain the abundance of this beneficial genus under inflammatory challenge.

*Megasphaera*, often associated with fermentative imbalances, exhibited a sharp ninefold increase in the LPS group compared to control (*p* < 0.001), indicating a strong dysbiotic response. The SYN+LPS group also showed a 3-fold increase versus control (*p* < 0.01), suggesting that the synbiotic diet stimulated *Megasphaera* growth, though to a slightly lesser extent than LPS. In this group, *Megasphaera* abundance was reduced by 27.5% compared to the LPS group (*p* > 0.05), indicating a partial mitigation effect.

*Lachnospira* increased 2.6-fold (Figure 5) in the LPS group compared to control (*p* < 0.001), again reflecting a dysbiotic shift. Conversely, the SYN group had a 65.4% reduction in *Lachnospira* relative to control (*p* < 0.001), indicating that the feed ingredient suppressed the genus under normal conditions. In the SYN+LPS group, *Lachnospira* abundance decreased by 2.7-fold compared to the LPS group (*p* < 0.05), suggesting a significant modulation of the LPS-induced elevation.

*Prevotella* (as a phylogenetic group constituted by *Alloprevotella*, *Prevotella_09*, *Prevotella_07* and *Prevotellaceae_NK3B31_group*) showed no significant differences in relative abundance across groups, with minor fluctuations that were not statistically meaningful (*p* > 0.05), indicating this genus remained relatively stable regardless of treatment.

**Figure 6:**
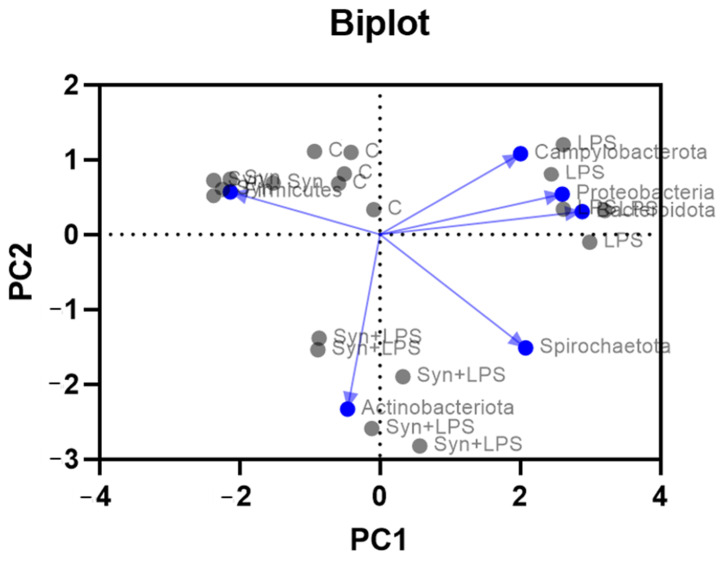
Biplot of microbiota genus composition in piglets across dietary groups. Each gray dot represents a sample within a dietary experimental group: C—control group, LPS—LPS group, SYN—synbiotic group, SYN+LPS—synbiotic with LPS. The first two ordination axes explain 42% (PC1) and 37% (PC2) of the variance, respectively. Blue dots with arrows represent key microbial genera (*Lactobacillus*, *Megasphaera*, *Faecalibacterium*, and *Lachnospira*), with the arrow direction indicating the association of each genus with the principal components. Gray dots represent individual piglet samples.

The principal coordinates analysis (PCoA) (Figure 6) based on Bray–Curtis distances revealed distinct microbial profiles across treatments. PC1 and PC2 explained 42% and 37% of the total variance, indicating that the two-dimensional projection summarizes most of the multivariate signal (79%). The control group formed a compact cluster, reflecting low variability and consistent microbial composition. In contrast, the LPS group displayed high dispersion, indicating substantial inter-individual variability and microbiota disruption. The SYN+LPS group showed a distinct, well-defined cluster, representing a treatment-specific microbial profile.

In the PCA biplot, the LPS group clustered separately on the positive side of PC1, indicating a marked shift in microbial composition relative to the other treatments. By contrast, the control, SYN and SYN+LPS samples all clustered in the left (negative PC1) quadrant, with SYN+LPS positioned between the control and SYN weight centers. This pattern suggests that the synbiotic co-treatment attenuated the LPS-induced divergence in community structure, maintaining a configuration closer to control and SYN than to LPS alone (Figure 6). These results indicate that the novel dietary treatment exerted a stabilizing effect in the presence of inflammatory challenge.

## 4. Discussion

Controlling weaning-associated intestinal disturbances remains a central objective in pig production because early mucosal injury predisposes piglets to poor growth and disease [30].

Nutritional strategies using synbiotics are being actively explored to limit intestinal barrier dysfunction, as well as the dysbiosis triggered by weaning stress and endotoxin exposure [31]. In this context, we evaluated a diet containing a synbiotic combination of grape seed and camelina meal prebiotics and a *Lactobacillus* mixture probiotic as a modulator of *Escherichia coli* LPS-induced disturbances in the colon of weaned piglets. The synbiotic diet was tested in healthy and LPS-challenged animals to determine its capacity to maintain intestinal barrier integrity and functionality, stabilize the microbiota, and preserve epithelial defense.

In this acute-LPS-challenge model, synbiotic supplementation was associated with a coordinated host–microbiota response, including transcriptomic signatures consistent with improved barrier function and the modulation of innate immune signaling, as well as changes in microbiota composition. Accordingly, we interpret these findings as evidence of intestinal molecular and microbial responses to the synbiotic diet under *E. coli* LPS challenge.

In a previous paper, we demonstrated that the SYN diet was able to prevent intestinal inflammation induced by LPS challenge in weaned piglets [25], leading to a restoration of intestinal integrity and functionality. An increased number of studies demonstrated that intestinal homeostasis is affected both by inflammation and by an impaired innate immune response [23,32,33,34].

In contrast to our previous publication, the present study expands the work by integrating high-throughput 16S rRNA gene profiling of the colon microbiota with a broader epithelial-/innate-immune-gene-expression panel assessed in the same animals. This combined host–microbiota dataset provides additional insight into how synbiotic supplementation is associated with concurrent shifts in microbial community structure and mucosal immune-barrier signaling, thereby extending beyond our earlier report and clarifying the novel contribution of the current manuscript.

The literature data showed that LPS treatment affected the integrity of intestinal epithelial cells and tight-junction protein expression in both in vitro and in vivo models [35,36,37]. As expected, in our study, the administration of LPS was associated with a reduction in the expression of genes coding for epithelial tight-junction and mucus-controlling proteins, including *CLDN2*, *CLDN4*, *CLDN14*, *CLDN20*, *OCLN*, and *ZO-1* in the colon, consistent with intestinal barrier impairment reported in post-weaning piglets [38]. Synbiotic administration mitigated the negative effects of the LPS challenge on intestinal epithelial integrity, as reflected by the increased expression of tight-junction-related genes in the colon. This pattern agrees with previous reports showing that synbiotic combinations can reinforce barrier function across both in vitro and in vivo models, most commonly through restoration or upregulation of key tight-junction components such as *ZO-1* and occludin (*OCLN*). For example, synbiotic formulations have been shown to improve epithelial barrier properties in challenged Caco-2 cell systems and to increase *ZO-1/OCLN* expression in animal models of intestinal inflammation, [34,39,40] while similar effects on tight-junction genes and proteins have also been reported in LPS-challenged weaned piglets receiving synbiotic supplementation [31,41].

The mucosal barrier is another important target triggered by LPS from Gram-negative bacteria [42]. Changes in the expression of the *MUC2* gene (encoding the major intestinal mucin) are being registered in LPS-challenged mice [43,44]. Similarly, in our experiment, the *MUC2* gene was reduced (a 35% decrease in the fold change relative to the control group) in the colon of LPS-challenged piglets and the effect restored by the SYN diet. In a cellular model using the MUC-2-secreting cell line HT-29, a synbiotic mix of seaweed fucoidan and *Lactobacillus plantarum* improved mucin production in dextran sodium-sulphate (DSS)-treated HT-29 cells [45].

The effects on tight-junction regulators were moderated: the relative stability of *MAGI2* after LPS challenge, together with its moderate increase under synbiotic administration, suggests a structurally robust scaffolding role with a modest synbiotic-mediated reinforcement of epithelial barrier resilience. The marked LPS-induced downregulation of *GNAI2*, and its normalization or enhancement with synbiotic treatment, highlights this regulator as a key target through which the synbiotic diet preserves epithelial polarity and barrier signaling. By contrast, the only partial recovery of *MYO9B* in the SYN+LPS group indicates that the cytoskeleton–junction coupling remains a vulnerable component, suggesting that myosin IXB-dependent support is less amenable to full restoration under the present synbiotic regimen.

**Figure 7:**
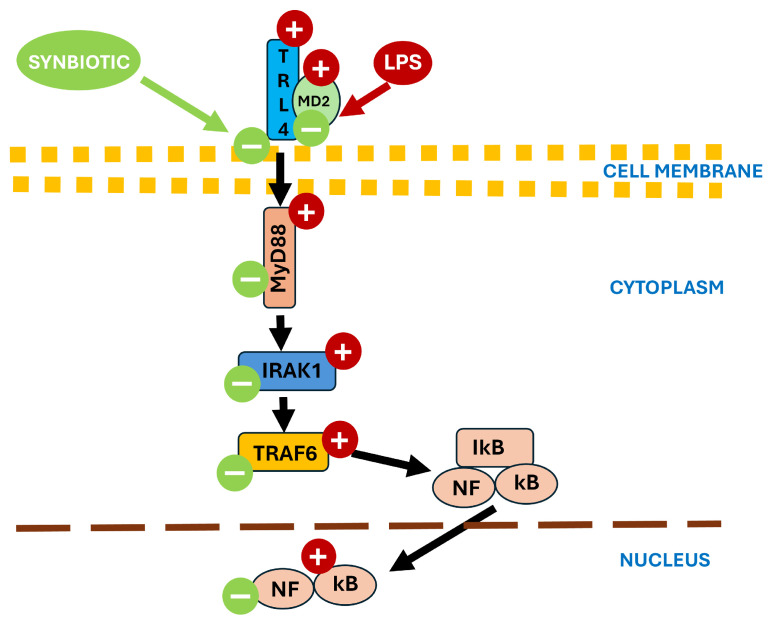
Proposed mechanism of action for the synbiotic mix, wherein LPS is an abbreviation for lipopolysaccharide; TLR4—Toll-like receptor 4; MD2—myeloid differentiation factor 2 (LY96); MyD88—myeloid differentiation primary response 88; IRAK1—interleukin-1 receptor associated kinase 1; TRAF6—TNF receptor associated factor 6; IκB—inhibitor of NF-κB; NF-κB—nuclear factor kappa B.

It was demonstrated that *E. coli* LPS acts on the intestinal epithelium in multiple ways, among them the increase in intestinal permeability by tight junctions’ disruption and the activation of TLR4 signaling via *NF-κB*, a canonical driver of pro-inflammatory cytokine production in the gut [36,46]. Indeed, in our study, the LPS challenge upregulated pattern-recognition receptors TLRs, with 70% of all analyzed TLR genes affected. These results are in accordance with previous studies on mice [47,48], which also showed that LPS affected TLR-associated signaling, with increased transcripts of M2/MyD88 and associated downstream molecules triggering the activation of *NF-κB*. In our study the synbiotic diet prevented these effects, dampening *TLR4-MyD88* activation in the colon of LPS-challenged piglets. These changes indicate a dual-action effect of the SYN diet in LPS-challenged piglets, by the strengthening of epithelial defenses as well as by the suppression of LPS endotoxin-driven signaling, consistent with prior evidence for *Lactobacillus* spp. and polyphenol-rich feeds in swine and rodent intestines [49]. Figure 7 illustrates a possible mechanism of action of the synbiotic.

Moreover, inflammation triggered through TLR4 activation has been associated with changes in gut microbiota composition [50]. In our study, microbiota-relative abundance aligned with the host response. Herein, LPS reduced alpha diversity and shifted community structure, features commonly associated with inflammatory stress and impaired ecological resilience in the weaning period. Given the study design, these findings do not allow us to determine whether the microbiota changes are primary drivers or secondary consequences of epithelial and inflammatory signaling, only that they are associated.

Synbiotic feeding preserved phylogenetic richness and limited between-group divergence, maintaining microbial communities closer to the control profile. At the phylum level, LPS disturbed the Firmicutes:Bacteroidota balance and increased Proteobacteria, a pattern which could suggest the presence of dysbiosis in pigs [49] nevertheless such phylum-level ratios are context-dependent and should not be interpreted as universal markers of dysbiosis. The synbiotic diet restored a more balanced phylum distribution and curtailed Proteobacteria expansion under challenge, consistent with a reduction in inflammatory tone.

Genus-level changes provide a finer-resolution view of the microbiota response. LPS reduced *Lactobacillus* and *Faecalibacterium* and increased genera associated with weaning stress, including *Megasphaera* and *Lachnospira*. The synbiotic diet reversed these shifts, restoring *Lactobacillus* and *Faecalibacterium* toward control levels and tempering the LPS-associated increases in stress-related genera, with similar results shown in the research literature [48]. These taxonomic changes coincided with preserved alpha diversity and a community configuration closer to the healthy control profile, and they the paralleled recovery of epithelial transcripts (*OCLN*, *ZO-1*, *CLDN2/CLDN4*, *MUC2*) together with the attenuation of *TLR4-MyD88* activation.

These protective effects of the SYN diet could be attributed to the enhancement of the gene expression of proteins involved in tight-junction formation, barrier integrity and function, positive regulation of TLRs, MD2/MyD88 and *NF-κB* signaling, as well as beneficial modulation of colon microbiota and suppression of gut pathogens.

The genus-level composition under the synbiotic diet resembled the healthy profile and mitigated LPS-associated dysbiosis, aligning with other readouts that indicate lower inflammatory activation in the intestinal mucosa. This pattern aligns with prior reports in LPS-challenged piglets by Miao et al. and Zheng et al. [31,51].

The combined prebiotic–probiotic formulation offers plausible routes to these effects. Grape seed and camelina meals supply bioactive compounds (e. g. fermentable fiber, unsaturated fatty acids, polyphenols, etc.) that selectively favor saccharolytic consortia and yield bioactive phenolics after microbial metabolism [52]. *Lactobacillus* strains can lower luminal pH, which can compete with pathogenic bacteria and interfere with endotoxin signaling by affecting ligand availability and *TLR4* co-receptor interactions [53]. Polyphenols and *Lactobacillus* metabolites have also been shown to constrain *MAPK* and *NF-κB* cascades and to modulate epithelial renewal pathways, providing a molecular rationale for the observed reduction in *TLR4-MyD88* signaling and improvement in barrier-gene expression [8,54]. Together, these substrate–microbe and microbe–host interactions present a coherent explanation for the convergence of microbial and epithelial readouts in the synbiotic groups.

However, we acknowledge that the study outcomes were constrained by limitations in the number of animals per group. This may limit statistical power to detect small effect sizes and increases the risk of overlooking subtle differences. Accordingly, larger cohorts would be beneficial in future studies to confirm and refine the magnitude of effects and to improve generalizability. Nevertheless, several of the differences observed in the present work were consistent, suggesting effects of sufficient magnitude to be detected despite the limited sample size. Accordingly, the underlying relationships proposed here should be interpreted as associative, due to the present design not differentiating between the probiotic component and the polyphenol/PUFA-rich fraction.

From a practical standpoint, these findings support synbiotics as a non-antibiotic tool to buffer weaning stress (e.g., bacterial infections). With the phaseout of prophylactic antimicrobials and pharmacological zinc, diets that pair agro-industrial by-products rich in bioactives and robust *Lactobacillus* strains may help stabilize the gut ecosystem and reduce the burden of enteric disorders in the nursery [55]. The present data extend previous reports by demonstrating the simultaneous modulation of community diversity, key phyla and genera, TLR-MyD88-axis signaling, and barrier-related transcripts within a single experiment. Given the limited sample size and the post-challenge sampling window, the present results should be interpreted as evidence of intestinal responses within this experimental framework.

## 5. Conclusions

In conclusion, our results demonstrated that LPS challenge in piglets after weaning produced a marked disturbance in the colon of piglets characterized by the reduced gene expression of tight-junction, mucus and Toll-like receptor proteins, activation of the *TLR4*-*NF-κB* pathway, and decreased microbial richness, as well as an imbalanced phylum profile with the expansion of stress-associated taxa and depletion of beneficial genera. The synbiotic diet including a mix of prebiotics (grape seed and camelina meals) and probiotic *Lactobacillus* strains could be an efficient dietary solution in the prevention of LPS endotoxin-induced intestinal barrier dysfunction and dysbiosis and may help alleviate post-weaning enteric problems in piglets.

These results support the use of synbiotic diet as a feasible strategy to reduce pathogen stress immediately after weaning and warrant further validation with functional and performance endpoints in production-relevant settings. Further studies a larger number of animals will be able to detect other subtle changes and assess their significance.

## Figures and Tables

**Table 1:** Relative expression of genes assuring intestinal epithelial barrier function in the colon of weaned piglets.

Gene	Experimental Groups *				
	Control (Fold Change)	LPS (Fold Change)	SYN (Fold Change)	SYN+LPS (Fold Change)	*p*-Value
*CLDN1*	1.00 ± 0.00 ^a^	0.79 ± 0.17 ^a^	4.31 ± 0.70 ^b^	1.17 ± 0.25 ^c^	0.007
*CLDN2*	1.00 ± 0.00 ^a^	0.14 ± 0.10 ^b^	2.09 ± 0.40 ^a^	1.21 ± 0.20 ^a^	0.009
*CLDN4*	1.00 ± 0.00 ^a^	0.62 ± 0.10 ^b^	3.90 ± 0.50 ^c^	1.24 ± 0.20 ^a^	0.008
*CLDN5*	1.00 ± 0.00 ^a^	0.74 ± 0.13 ^a^	0.50 ± 0.12 ^b^	0.45 ± 0.04 ^a^	0.048
*CLDN14*	1.00 ± 0.00 ^a^	0.46 ± 0.03 ^b^	2.76 ± 0.78 ^a^	1.07 ± 0.16 ^a^	0.009
*CLDN20*	1.00 ± 0.00 ^a^	0.33 ± 0.03 ^b^	6.09 ± 0.60 ^c^	0.73 ± 0.20 ^d^	0.001
*CLDN23*	1.00 ± 0.00 ^a^	0.81 ± 0.05 ^a^	2.59 ± 0.20 ^b^	0.88 ± 0.08 ^a^	0.041
*OCLN*	1.00 ± 0.00 ^a^	0.62 ± 0.10 ^b^	2.20 ± 0.20 ^c^	1.91 ± 0.10 ^c^	0.007
*ZO-1*	1.00 ± 0.00 ^a^	0.64 ± 0.10 ^b^	7.17 ± 0.50 ^c^	1.14 ± 0.40 ^a^	0.001
*JAM-A*	1.00 ± 0.00 ^a^	0.57 ± 0.06	2.39 ± 0.24	1.06 ± 0.20	0.008
*MUC2*	1.00 ± 0.00 ^a^	0.65 ± 0.10 ^b^	2.56 ± 0.30 ^c^	1.44 ± 0.10 ^c^	0.007
*ECM1*	1.00 ± 0.00 ^a^	0.74 ± 0.20 ^a^	1.57 ± 0.10 ^b^	0.98 ± 0.10 ^a^	0.042

* Post-weaned piglets were assigned to a control diet (control and LPS groups) or SYN diet (SYN and SYN+LPS groups) for 21 days. On day 21 of the experiment, piglets from the LPS and SYN+LPS groups were challenged with 80 µg/b.w. LPS. After 3 h, colon samples from all animals (n = 5) were collected and analyzed for gene expression by qPCR arrays; the results, expressed as fold changes (Fcs), are presented as mean ± SEM. The *p*-values reported in the last column correspond to the overall one-way ANOVA for each gene (row). ^a,b,c,d^ Different superscript letters within a row indicate significant differences (*p* < 0.05) according to Tukey’s post hoc test following one-way ANOVA.

**Table 2:** Expression of dietary treatments on tight-junction regulator genes in the colon of weaned piglets.

Gene	Experimental Groups *				
	Control (Fold Change)	LPS (Fold Change)	SYN (Fold Change)	SYN+LPS (Fold Change)	*p*-Value
*MAGI2*	1.00 ± 0.00	0.98 ± 0.22	1.41 ± 0.16	1.16 ± 0.17	n.s.
*GNAI2*	1.00 ± 0.00 ^a^	0.61 ± 0.05 ^b^	1.59 ± 0.10 ^c^	0.96 ± 0.10 ^a^	0.008
*MYO9B*	1.00 ± 0.00 ^a^	0.67 ± 0.05 ^b^	1.15 ± 0.05 ^a^	0.83 ± 0.01 ^b^	0.043

* Post-weaned piglets were assigned to a control diet (control and LPS groups) or SYN diet (SYN and SYN+LPS groups) for 21 days. On day 21 of the experiment, piglets from the LPS and SYN+LPS groups were challenged with 80 µg/b.w. LPS. After 3 h, colon samples from all animals (n = 5) were collected and analyzed for gene expression by qPCR arrays; the results, expressed as fold changes (Fcs), are presented as mean ± SEM. The *p*-values reported in the last column correspond to the overall one-way ANOVA for each gene (row). ^a,b,c^ Different superscript letters within a row indicate significant differences (*p* < 0.05) according to Tukey’s post hoc test following one-way ANOVA (n.s. = non-significant).

**Table 3:** The effects of dietary treatments on the relative expression of Toll-like receptor genes in the colon of weaned piglets.

Gene	Experimental Groups *				
	Control (Fold Change)	LPS (Fold Change)	SYN (Fold Change)	SYN+LPS (Fold Change)	*p*-Value
*TLR1*	1.00 ± 0.00 ^a^	2.10 ± 0.20 ^b^	1.02 ± 0.26 ^a^	0.97 ± 0.19 ^a^	0.036
*TLR2*	1.00 ± 0.00 ^a^	1.93 ± 0.10 ^b^	1.47 ± 0.40 ^a^	1.29 ± 0.20 ^a^	0.039
*TLR3*	1.00 ± 0.00 ^a^	1.51 ± 0.13 ^b^	1.21 ± 0.21 ^a^	0.76 ± 0.18 ^a^	0.045
*TLR4*	1.00 ± 0.00 ^a^	2.74 ± 0.10 ^b^	0.93 ± 0.20 ^a^	0.71 ± 0.10 ^c^	0.007
*TLR5*	1.00 ± 0.00 ^a^	1.57 ± 0.24 ^b^	1.40 ± 0.27 ^a^	0.76 ± 0.06 ^a^	0.046
*TLR6*	1.00 ± 0.00 ^a^	2.12 ± 0.10 ^b^	0.69 ± 0.10 ^a^	0.75 ± 0.30 ^a^	0.037
*TLR7*	1.00 ± 0.00 ^a^	1.81 ± 0.17 ^b^	0.95 ± 0.21 ^a^	0.74 ± 0.15 ^a^	0.038
*TLR8*	1.00 ± 0.00 ^a^	1.87 ± 0.10 ^b^	1.05 ± 0.10 ^a^	0.70 ± 0.10 ^a^	0.039
*TLR9*	1.00 ± 0.00	1.21 ± 0.17	0.98 ± 0.11	1.32 ± 0.12	n.s.
*TLR10*	1.00 ± 0.00 ^a^	2.73 ± 0.35 ^b^	1.66 ± 0.47 ^a^	0.94 ± 0.16 ^a^	0.031

* Post-weaned piglets were assigned to a control diet (control and LPS groups) or SYN diet (SYN and SYN+LPS groups) for 21 days. On day 21 of the experiment, piglets from the LPS and SYN+LPS groups were challenged with 80 µg/b.w. LPS. After 3 h, colon samples from all animals (n = 5) were collected and analyzed for gene expression by qPCR arrays; the results, expressed as fold changes (Fcs), are presented as mean ± SEM. The *p*-values reported in the last column correspond to the overall one-way ANOVA for each gene (row). ^a,b,c^ Different superscript letters within a row indicate significant differences (*p* < 0.05) according to Tukey’s post hoc test following one-way ANOVA; n.s. = non-significant.

**Table 4:** The effects of dietary treatments on the relative expression of signaling mediator genes in the colon of weaned piglets.

Gene	Experimental Groups *				
	Control (Fold Change)	LPS (Fold Change)	SYN (Fold Change)	SYN+LPS (Fold Change)	*p*-Value
*MD2*	1.00 ± 0.00 ^a^	3.75 ± 0.30 ^b^	0.91 ± 0.11 ^a^	1.11 ± 0.63 ^a^	0.027
*MyD88*	1.00 ± 0.00 ^a^	6.70 ± 0.84 ^b^	1.13 ± 0.20 ^a^	1.98 ± 0.32 ^c^	0.004
*IRAK1*	1.00 ± 0.00 ^a^	3.51 ± 0.54 ^b^	0.65 ± 0.26 ^c^	0.92 ± 0.17 ^a^	0.009
*TRAF6*	1.00 ± 0.00 ^a^	2.08 ± 0.26 ^b^	2.10 ± 0.36 ^b^	0.52 ± 0.07 ^c^	0.005
*IkB*	1.00 ± 0.00 ^a^	1.07 ± 0.06 ^a^	4.03 ± 0.54 ^b^	0.78 ± 0.12 ^c^	0.004
*NF-κB/p65*	1.00 ± 0.00 ^a^	2.34 ± 0.26 ^b^	0.28 ± 0.01 ^c^	0.61 ± 0.15 ^d^	0.001

* Post-weaned piglets were assigned to a control diet (Control and LPS groups) or SYN diet (SYN and SYN+LPS groups) for 21 days. On day 21 of the experiment, piglets from the LPS and SYN+LPS groups were challenged with 80 µg/b.w. LPS. After 3 h, colon samples from all animals (n = 5) were collected and analyzed for gene expression by qPCR arrays; the results, expressed as fold changes (Fcs), are presented as mean ± SEM. The *p*-values reported in the last column correspond to the overall one-way ANOVA for each gene (row). ^a,b,c,d^ Different superscript letters within a row indicate significant differences (*p* < 0.05) according to Tukey’s post hoc test following one-way ANOVA.

## Data Availability

The original contributions presented in this study are included in the article and Supplementary Material. Further inquiries can be directed to the corresponding author.

## Supplementary Materials

The following supporting information can be downloaded at https://www.mdpi.com/article/10.3390/cimb48030298/s1.
