# Supplementary material for: Synbiotic Diet Prevents Escherichia coli Lipopolysaccharide-Induced Gut Dysbiosis and Intestinal Disruption After Weaning in Piglets
Source: Curr Issues Mol Biol. 2026 Mar 11;48(3):298. doi: 10.3390/cimb48030298 (PMC13025845; doi:10.3390/cimb48030298)
Supplement: Supplementary file 1 [file cimb-48-00298-s001.zip › Synbiotic diet prevents LPS 2026 supplementary.pdf]

Supplementary Table S1. List and characteristics of target genes detected in qPCR array

No crt	Species	Accession No	Gene abbreviation	Gene name	qPCR amplicon size (bp)
1	Sus scrofa	NM_001031775.1	*TLR1*	Toll-like receptor - 1	153
2	Sus scrofa	NM_213761.1	*TLR2*	Toll-like receptor - 2	153
3	Sus scrofa	NM_001097444.1	*TLR3*	Toll-like receptor - 3	109
4	Sus scrofa	NM_001113039.2	*TLR4*	Toll-like receptor - 4	159
5	Sus scrofa	NM_001348771.1	*TLR5*	Toll-like receptor - 5	99
6	Sus scrofa	NM_213760.2	*TLR6*	Toll-like receptor -6	97
7	Sus scrofa	NM_001097434.1	*TLR7*	Toll-like receptor - 7	92
8	Sus scrofa	NM_214187.1	*TLR8*	Toll-like receptor - 8	170
9	Sus scrofa	NM_213958.1	*TLR9*	Toll-like receptor - 9	100
10	Sus scrofa	NM_001030534.1	*TLR10*	Toll-like receptor - 10	139
11	Sus scrofa	NM_001105286.1	*TRAF-6*	TNF Receptor Associated Factor 6	160
12	Sus scrofa	XM_003135490.4	*IRAK1*	Interleukin 1 Receptor Associated Kinase 1	159
13	Sus scrofa	NM_001099935.1	*IkB*	Inhibitor of kappa B	151
14	Sus scrofa	NM_001114281.1	*NF-kB/p65*	Nuclear Factor Kappa B	110
15	Sus scrofa	NM_001099923.1	*MyD88*	Myeloid differentiation primary response 88	124
16	Sus scrofa	NM_001104956.1	*MD-2*	Myeloid differentiation factor 2	140
17	Sus scrofa	NM_001244539.1	*CLDN1*	Claudin-1	95
18	Sus scrofa	NM_001161638.1	*CLDN2*	Claudin-2	95
19	Sus scrofa	NM_001161637.1	*CLDN4*	Claudin-4	106
20	Sus scrofa	NM_001161636.1	*CLDN5*	Claudin-5	152
21	Sus scrofa	NM_001161642.1	*CLDN14*	Claudin-14	99
22	Sus scrofa	NM_001159777.1	*CLDN20*	Claudin-20	140
23	Sus scrofa	NM_001159778.1	*CLDN23*	Claudin-23	104
24	Sus scrofa	NM_001163647.2	*OCLN*	Occludin	155
25	Sus scrofa	XM_021098845.1	*ZO-1*	Zonula-1	109
26	Sus scrofa	XM_021066740.1	*JAM-A*	Junctional adhesion molecule A	119
27	Sus scrofa	XM_021094903.1	*MAGI2*	Membrane Associated Guanylate Kinase, WW And PDZ Domain Containing 2	139
28	Sus scrofa	XM_003356615.4	*GNAI2*	G Protein Subunit Alpha I2	93
29	Sus scrofa	NM_001130211.1	*MYO9B*	Myosin IX B	165
30	Sus scrofa	XM_021082584.1	MUC2	Mucin-2	169
31	Sus scrofa	XM_021089906.1	ECM1	Extracellular matrix protein	158
